# Post-mortem evaluation of intervertebral disc degeneration using magnetic resonance imaging and magnetisation transfer ratio in chondrodystrophic and non-chondrodystrophic dogs: a pilot study

**DOI:** 10.1371/journal.pone.0329884

**Published:** 2026-04-21

**Authors:** Charlène Paquet, Arnaud Lefeuvre, Julien Fritz, Laurent Couturier, Rémi Bellon, Charles Montel, Nicolas Gaide, Germain Arribarat, Giovanni Mogicato

**Affiliations:** 1 Université de Toulouse, Toulouse, France; 2 ENVT, Toulouse, France; 3 Azurvet, Centre de vétérinaires spécialistes, Saint-Laurent-du-Var, France; 4 ToNIC, Toulouse NeuroImaging Center, Université de Toulouse, INSERM, UPS, ENVT, Toulouse, France; 5 IHAP, Université de Toulouse, INRAe, ENVT, Toulouse, France; 6 ToNIC, Toulouse NeuroImaging Center, Université de Toulouse, INSERM, UPS, Toulouse, France; Museo Storico della Fisica e Centro Studi e Ricerche Enrico Fermi, ITALY

## Abstract

Intervertebral disc degeneration is a frequent and early condition in chondrodystrophic dogs, which can lead to neurological disorders due to compression of the spinal cord. The symptoms include pain, reduced mobility and even paralysis. Magnetic resonance imaging (MRI) is currently the reference technique for analysing this pathology. Based on T2-weighted MRI sequences, Pfirrmann’s classification provides a qualitative assessment of the evolution of disc lesions. However, this approach remains subjective and subject to inter-observer variability, underscoring the need for more objective and reproducible tools. The aim of this pilot study was to explore the value of the magnetization transfer ratio (MTR) as a quantitative tool for a more accurate assessment of disc degeneration. MRI acquisitions were performed on spinal columns harvested post-mortem from 9 chondrodystrophic and non-chondrodystrophic dogs of various breeds. 181 intervertebral discs were graded according to the Pfirrmann scale, and MTR values were measured within the nucleus pulposus, annulus fibrosus and the entire disc. Moreover a correlation between imaging findings, macroscopic evaluation with Thompson grade and histological staining was made to ensure accurate assessment of intervertebral disc degeneration. Statistical analysis was carried out to examine the correlation between these values and the degree of disc degeneration. The results show a positive correlation between increased MTR and the severity of Pfirrmann grades. These data suggest that MTR MRI could provide a complementary tool for more reliable and standardised quantification of disc degeneration, reducing subjective bias. This approach could improve diagnostic accuracy and early assessment of disc alterations, with important implications for therapeutic management. Nonetheless, an *in vivo* validation of this approach remains necessary.

## 1. Introduction

The intervertebral disc (IVD), consisting of a central nucleus pulposus (NP) surrounded by an annulus fibrosus (AF), is a key structure in the spinal column. Placed between two vertebrae, it absorbs shock, keeps the spine mobile and helps protect the spinal cord [1,2].

With age, all anatomical structures, including those of dogs, are subject to progressive deterioration, and the spine is no exception. This degenerative process is a common condition, but it occurs early in so-called chondrodystrophic (CD) dogs, a population with a genetic skeletal abnormality affecting cartilage development [3,4]. IVD degeneration in CD dogs begins as early as three months of age and is located first in the NP and then the AF. In contrast, in non-chondrodystrophic dogs (NCD), it occurs later and progresses in a progressive manner, affecting both the NP and the AF [5,6].

Magnetic resonance imaging (MRI) is considered to be the reference method for assessing IVD degeneration thanks to its excellent tissue contrast resolution [2,7]. Initially developed for human medicine, the Pfirrmann classification enables disc degeneration to be graded qualitatively from I to V on T2-weighted sequences [8]. This method, recently transposed to veterinary medicine, has shown good correlation with histopathological observations, particularly on the basis of signal intensity and disc height [7]. However, there is some inter-observer subjectivity and limitation to reproducibility.

In human medicine, quantitative approaches have been developed, notably using the MTR (Magnetization Transfer Ratio) sequence in MRI. This measures magnetisation exchanges between free hydrogen protons and those bound to macromolecules such as collagen by using a staggered radiofrequency pulse. Collagen has been shown to be responsible for a significant magnetisation transfer effect [9,10].

Collagen is the main macromolecule in AF. It is also found in smaller proportions in NP. Before the age of one year, the NP of CD breeds contains around 25% collagen, compared to less than 5% in NCD breeds. The collagen content of NP and AF is therefore significantly higher in CD dogs than in NCD dogs [5].

It has been shown, using a canine model that early degenerative or traumatic IVD changes are detectable with T2WI and MTR imaging and correlate well with histopathological findings [11]. In addition, a human study demonstrated a moderate linear correlation between MTR values and Pfirrmann grades, suggesting an increase in relative collagen density within the NP as degeneration progresses [10]. Recently, these observations have been extended to veterinary medicine in chondrodystrophic dogs. One study showed a positive, moderate and significant correlation between the Pfirrmann score and MTR values, indicating an increase in magnetisation transfer in the NP as disc degeneration progresses [12]. These results suggest that MTR MRI provides an objective method of quantitative assessment for detecting early degeneration of IVDs. However, this study focused exclusively on the disc as a whole and on a single chondrodystrophic breed.

The aim of the present study was therefore to assess the correlation between the Pfirrmann degeneration score and MTR values in CD and NCD dogs. Three distinct regions will be analysed: the global intervertebral disc, the nucleus pulposus and the annulus fibrosus.

The interest of this study lies in the potential of MTR imaging to provide a more objective and region-specific assessment of disc degeneration. By comparing CD and NCD dogs and differentiating anatomical substructures, this approach could refine the assessment of degenerative changes and improve the early diagnosis of intervertebral disc disease.

## 2. Materials and methods

This study was a prospective comparative post-mortem randomised study. The experimental protocol and euthanasia conditions were approved by the Ethics Committee “Sciences et Santé Animale 115 - Ecole Nationale Vétérinaire de Toulouse” under the authorization number APAFIS#21559-2019071917392588v3.

All animals included in this study were euthanized for reasons unrelated to this research. The dogs included were client-owned animals whose owners had signed an informed consent form. Euthanasia was performed by licensed veterinarians using an overdose of intravenous barbiturates following deep sedation, in accordance with current European and national animal welfare regulations and with ethical guidelines to minimize pain and distress. After euthanasia, the animals were transferred to the pathology department of the Toulouse Veterinary School.

No anesthesia or analgesia procedures were performed as part of this research, since all samples were collected post-mortem. All imaging and macroscopic/histological evaluations were conducted exclusively on cadaveric specimens. Because the study involved only post-mortem material, no live animal underwent any procedure, and therefore no measures for alleviating suffering were required.

The vertebral columns were harvested from cadavers and frozen at −20°C for approximately 2 months. Four days before MRI acquisition, the spinal columns were placed in a cold room at 4°C for image acquisition to ensure gradual thawing. The imaging was carried out at the Purpan University Hospital Centre.

### 2.1. Image acquisition

The MRI images were taken using a Philips (Amsterdam, Netherlands) 3 Tesla MRI scanner, located in the 3T MRI technical platform of UMR 1214 ToNIC, Purpan (Toulouse, France). To replicate the sternal decubitus position, the spinal columns were oriented cranially toward the MRI bore, with the ventral side placed against the table. A 16-channel flexible elbow coil (Philips) was placed around the vertebral column. Images were acquired for the entire spine. T2-weighted images were obtained in the sagittal plane with the detailed MRI acquisition parameters shown in Table 1. Next, a magnetization transfer (MT) sequence was performed in the sagittal and transverse planes using the same slice placement as the T2-weighted sequence, with detailed acquisition parameters also shown in Table 1. MTR data were obtained using a dual-acquisition sagittal and transverse gradient echo sequence and collected with or without the application of a magnetisation transfer prepulse. One acquisition was performed in free water for 2.5 ms with an off-resonance pulse applied at 1000 Hz below the resonance frequency of the proton (MSAT) and the second acquisition was performed without the off-resonance pulse (M0). Finally, a third set of MTR images was calculated via a pixel-by-pixel algorithm using the formula: MTR = (M0-MSAT)/ M0. The value of the MTR signal was expressed as a saturation percentage (0–100%) calculated using the formula: MTR (%) = [(M0-MSAT)/ M0]x100.

**Table 1:** Image acquisition parameters for the T2-weighted images and for the magnetization transfer ratio (MTR) sequence.

Sequence	T2WI sagittal	MTR sagittal and transverse (M0)	MTR sagittal and transverse (MSAT)
Repetition time TR (ms)	3,000	72	72
Echo time TE (ms)	90	4,5	4,5
Angle flip (°)	18°	18°	18°
Matrix	400*400*16	400*400*16	400*400*16
Voxel size (mm)	0,7*0,7	0,7*0,7	0,7*0,7
Slice thickness (mm)	2	2	2
Interslice gap (mm)	0	0	0
Number of slices	16	16	16
Echo train (slice)	1	1	1
Acquisition number	3	3	3
Acquisition time (min)	3	27	27
Band width (Hz)	1,200	1,200	1,200
Pre-saturation impulsion RF	–	–	1,000 Hz for 2.5 ms

### 2.2. Qualitative analysis – Pfirmann Grading

The T2-weighted images were examined by six veterinary surgeons, graduates, associate members or residents of the European College of Veterinary Diagnostic Imaging (ECVDI), at the Azurvet clinic in Saint-Laurent-du-Var (06700), using Horos® software (3.0, Horos Project – Geneva, Switzerland). The specialists were as follows: R.D, F.J, C.E, C.L, A.F, B.R.

The image used for classification could differ, for the same vertebral column, depending on the area of interest studied. It was also optimally adjusted to display the disc and visualise the NP and AF. The observers classified the IVDs by consensus using the Pfirmann classification, ranging from I to V, to assess the degree of degeneration. This is based on several criteria, including T2 signal intensity, disc structure, distinction between nucleus pulposus and annulus fibrosus, and disc height. It is important to note that due to the post-mortem nature of the study, the comparison of NP and cerebrospinal fluid signal was not evaluable for grading.

**Fig 1:**
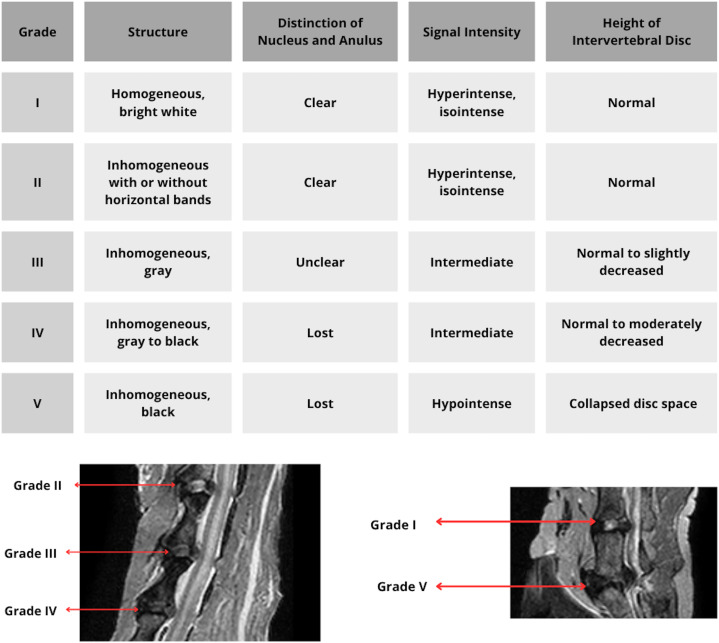
Pfirmann classification of disc degeneration: MRI characteristics of the intervertebral disc according to grades I to V, illustrated by MRI images from the study.

Grade I was assigned if the disc was homogeneous with an intense and bright signal, a distinction between NP and AF, and a normal disc cranio-caudal length; grade II, if the disc was inhomogeneous, but with a preserved intense signal and while maintaining a differentiation between NP and AF; grade III shows an inhomogeneous disc with a signal of intermediate intensity, the distinction between NP and AF less defined and a slightly decreased disc height; grade IV corresponds to a grey to black inhomogeneous structure with an intermediate to hypointense signal intensity, the distinction between NP and AF is absent, and the height of the IVD is moderately reduced; and grade V, if the structure is inhomogeneous with a hypointense signal, an absent distinction between NP and AF, and a reduced disc space (Fig 1).

### 2.3. Quantitative analysis – magnetization transfer ratio

For the comparative study, three regions of interest (ROI) were defined: the whole disc, the NP and the AF. These regions were defined using Sisyphe® neuroimaging software (Centre hospitalo-universitaire Purpan, Toulouse – France) by two observers: a medical imaging engineer and a veterinary surgeon based in Toulouse. ROIs were delimited in three planes: sagittal, transverse and coronal. Only the entire disc and the NP were contoured, then a subtraction operation was performed between these two volumes using the ‘exclusive OR between ROI volumes’ tool, to obtain a third region of interest corresponding solely to the AF [13,14]. Contouring of the NP and AF was carried out only when possible, depending on the grade (grade I to III).

A pooling step was carried out in order to retain only the areas of common interest for each region, with the aim of minimising the biases linked to differences in segmentation and guaranteeing greater reliability of the extracted measurements.

The ROIs were then saved and used on the corresponding MTR sequence. For each ROI, the following data was recorded: ROI volume, minimum MTR value (MTRmin), mean MTR value (MTRmean), median MTR value (MTRmed) and maximum MTR value (MTRmax).

### 2.4. Correlation between macroscopic grading, histological staining and Pfirmann grading

Since frozen spinal columns were used in this study, it is necessary to correlate our imaging findings (Pfirrmann grading) with macroscopic evaluation (Thompson grade) and histological staining to ensure accurate assessment of intervertebral disc degeneration.

A histological analysis was performed on a representative subset of four intervertebral discs in chondrodystrophic dogs with varying degrees of degeneration (ranging from grade I to grade IV according to Pfirrmann’s classification after examination by T2-weighted imaging). Histological analyses were intentionally limited to this representative subset and were used to provide qualitative biological context to MRI and macroscopic findings, rather than to serve as a quantitative validation across the entire dataset.

Frozen tissues were placed in 10% neutral buffered formalin for fixation for at least one week and subsequently decalcified in 10% EDTA solution (pH 7.4) at 37 °C for 10 days until tissue softening. Intervertebral discs were then cut transversely and grossly scored according to Thompson’s grading system [15]. Tissues were subsequently fixed again for an additional 48 h, embedded in paraffin, cut at 3 µm, and stained using a combined Picrosirius red and Alcian blue staining method. Slides were scanned at ×20 magnification using an Olympus VS200 slide scanner.

The determination of macroscopic grades and the interpretation of stained histological slides were evaluated by a veterinary surgeon (NG), graduate, associate member of the European College of Veterinary Pathologists (ECVP).

### 2.5. Comparative study – statistical analysis

Analyses were performed using commonly used software: Excel version 16.98 (25060824) and Prism 10 (version 10.4.2.633, GraphPad Software, USA). Two groups of samples were drawn up: one for CD breeds and the other for NCD breeds. Within each of these populations, subgroups were defined for each anatomical region: the whole disc, the NP and the AF.

First, the normality of the data was assessed. Then, a Pearson correlation was used to study the inter-operator variability for the volume of each ROI. The correlation between Pfirmann grades and MTR values was also examined using Pearson correlation coefficients. An analysis of variance (ANOVA) was used to compare the different grades within the same population and region. Finally, a Student’s t-test was used to compare chondrodystrophic and non-chondrodystrophic breeds in each region for the same grade.

## 3. Results

### 3.1. Study population

Nine vertebral columns were included in this study (Table 2). According to Dickinson’s and Brisson’s study, there were five CD dogs and four NCD dogs with a median age of 5 years and 1 month at the time of death. [2,16]. The dogs had no abnormalities on clinical or neurological examination. The number of IVDs visible on the MRI sequences varied for each dog. A total of 181 IVDs were analysed in this study (range: 15–25 IVD per dog).

**Table 2:** Study population: breed, age, gender and type.

Breed	Age	Gender	Type
Pug	5 months	Male	CD
Jack Russel Terrier	6 months	Male	CD
Beagle	5 years et 1 month	Female	CD
Cavalier King Charles	9 years and 11 months	Male	CD
Tulear cotton	17 years and 3 months	Male	CD
Belgian Malinois Shepherd	3 years	Male	NCD
Beauceron	4 years et 7 months	Female	NCD
Labrador Retriever	6 years et 9 months	Male	NCD
Border Collie	12 years et 5 months	Female	NCD

### 3.2. Macroscopic grading, histological staining, Pfirmann grading and MTR sequences

**Fig 2:**
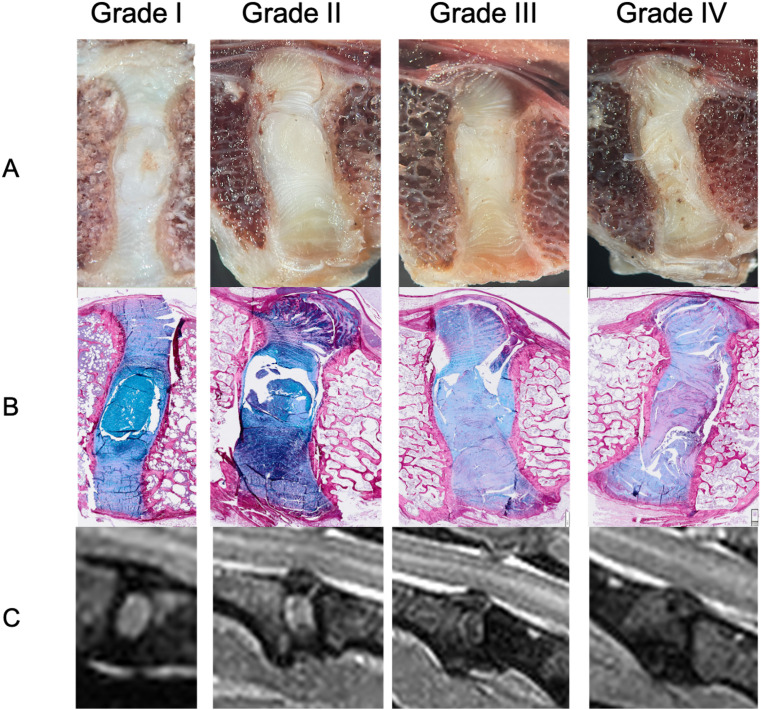
Corresponding macroscopic aspect (A), histological staining (Alcian blue and Picrosirius red staining) (B) and T2-weighted images (C) of intervertebral discs with degenerative changes (grade I to grade IV) according to Thompson’s grading system and Pfirmann’s grading system in chondrodystrophic dogs.

A good overall agreement was observed between the MRI-based Pfirrmann grading and the macroscopic Thompson grading of intervertebral discs (Fig 2). Discs classified as Pfirrmann grade I or II typically corresponded to Thompson grade I or II, reflecting a preserved disc structure with minimal degenerative changes. Higher Pfirrmann grades (III–IV) were associated with increased macroscopic signs of degeneration, including reduced disc height, loss of nucleus pulposus translucency, and annular fibrosus disorganization, corresponding to Thompson grades III–IV. Histological staining confirmed these findings, showing progressive loss of matrix organization and increased fibrous tissue content with higher degeneration grades. These correlations support the reliability of MRI grading in assessing intervertebral disc degeneration in frozen canine spines.

**Fig 3:**
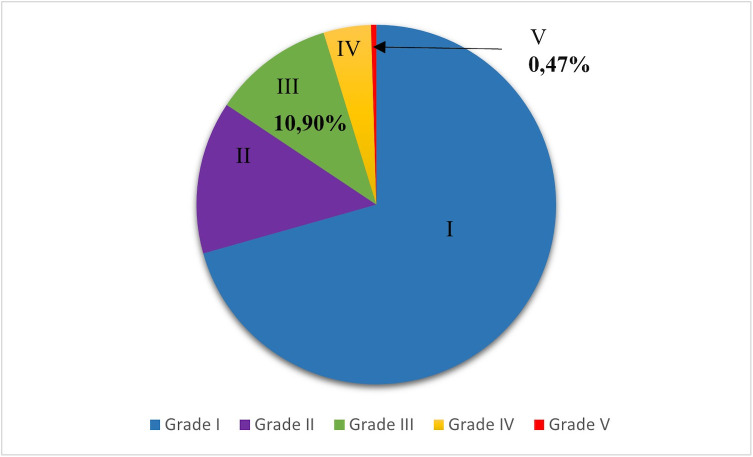
Proportion of different grades of chondrodystrophic and non-chondrodystrophic dogs.

Once the Pfirmann grades had been assigned, 125/181 were grade I (70.62%), 27/181 were grade II (13.74%), 21/181 were grade III (10.90%), 7/181 were grade IV (4.27%) and 1/181 was grade V (0.47%). The results are also shown in Fig 3 below.

**Fig 4:**
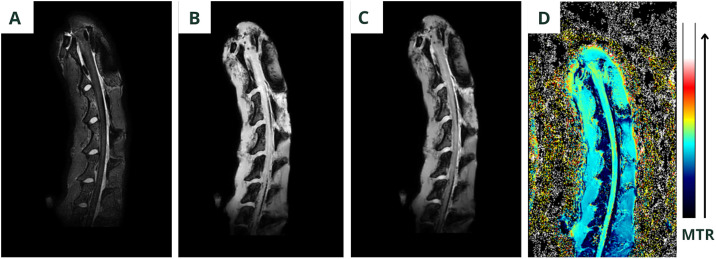
Different sets of images of the MTR sequence. (A) Image without the off-resonance RF (M0). (B) Image with the off-resonance saturation RF (MSAT). (C) Pixel-by-pixel computed MTR image (MTR = (M0 - MSAT)/ M0). (D) MTR color map image showing regional signal distribution.

Four types of images were obtained from the magnetization transfer sequences: images without the off-resonance RF with the signal M0, images with the off-resonance saturation RF with the signal MSAT, images calculated with the MTR pixel-by-pixel algorithm (MTR=(M0-MSAT)/M0) and images of the MTR color map. These images are displayed in Fig 4.

**Fig 5:**
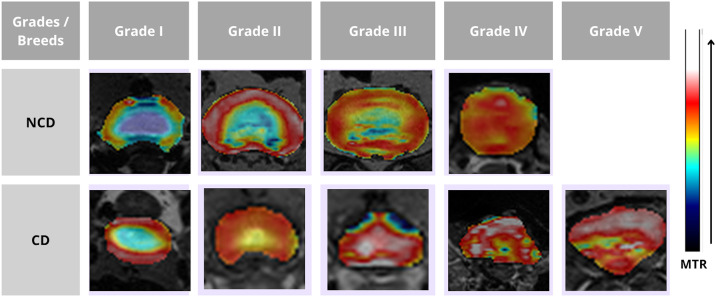
Overlay of the colour mapping of the MTR sequence on the reference T2 according to the different grades and the two populations.

Overlaying the colour maps of the MTR sequence on the T2 sequence shows the evolution of disc degeneration according to the five grades for the CD and NCD breeds (Fig 5). In grade I, the NP and AF are clearly distinguished with a low MTR value in the nucleus but a high value in the AF. Grade II is characterised by a homogeneous IVD structure and a less clear distinction between NP and AF. The AF always has a higher MTR value than the NP. Grade III shows a heterogeneous structure with a loss of sharpness between the NP and the AF. The NP begins to merge into the AF and the MTR value of the whole disc increases. Grades 4 and 5 shows a heterogeneous structure with no distinction between NP and AF; MTR values are high throughout the disc.

### 3.3. Statistical analysis

#### 3.3.1. Inter-operator variations.

**Fig 6:**
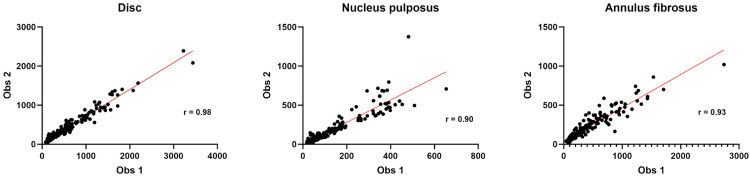
Scatter plots illustrating the delimitation of the IVD ROI between observers.

Pearson correlation coefficients for inter-operator agreement for ROI volume measurement for disc, NP and AF were respectively very good: 0.98 for disc, 0.90 for NP and 0.93 for AF (Fig 6).

#### 3.3.2.. Chondrodystrophic breeds.

**Fig 7:**
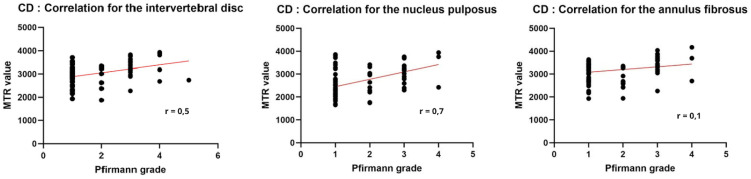
Pearson correlation between Pfirmann grade and MTR value in chondrodystrophic dogs.

Analysis of the correlation between MTR and Pfirrmann grades using Pearson’s coefficient reveals clear differences in CD dogs, as illustrated in Fig 7: a strong and significant positive correlation is demonstrated between MTR and Pfirrmann grade, particularly at the level of the NP (rho = 0.7) and for the whole intervertebral disc (rho = 0.5). However, no significant correlation was observed for the AF.

**Fig 8:**
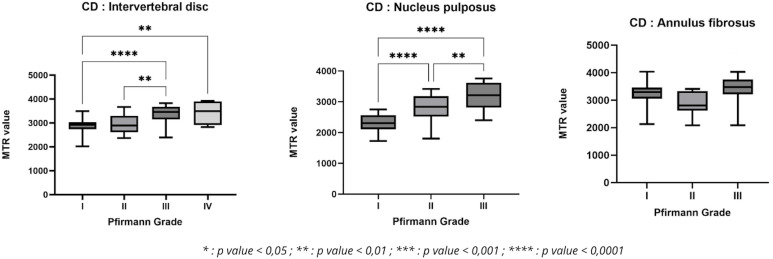
Variation in MTR value as a function of Pfirmann grade in chondrodystrophic dogs.

Statistical analysis using the ANOVA test revealed significant differences between certain Pfirmann grades in CD dogs. In the intervertebral disc, significant differences were observed between grades I/III, I/IV, and II/III. Concerning the NP, there were significant differences between grades I/II, I/III, and II/III. There were no significant differences for AF. (Fig 8).

#### 3.3.3. Non-chondrodystrophic breeds.

**Fig 9:**
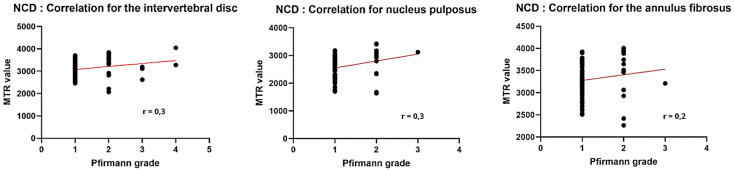
Pearson correlation between Pfirmann grade and MTR value in non-chondrodystrophic dogs.

In NCD dogs, the correlation between Pfirmann grades and MTR values was more moderate. It remains significant for the global intervertebral disc (rho = 0.30) as well as for the NP (rho = 0.29). Similarly, no significant correlation was observed for the AF (rho = 0.20) (Fig 9).

**Fig 10:**
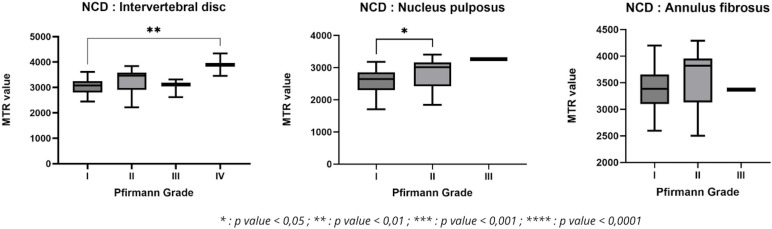
Variation in MTR value as a function of Pfirmann grade in non-chondrodystrophic dogs.

Significant difference was mainly observed in the NP between grades I/II, and in the disc between grades I/IV. On the other hand, no significant difference was found for AF (Fig 10).

#### 3.3.4. Comparison between the CD and NCD breeds.

**Fig 11:**
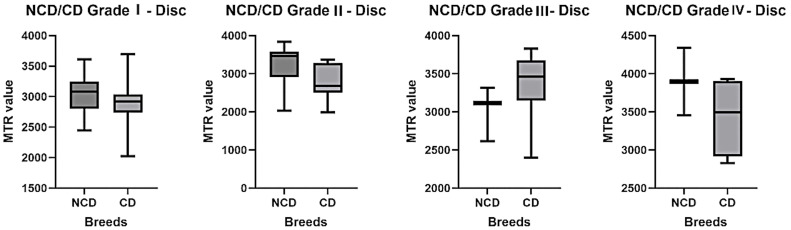
Variation of the MTR for the disc as a function of the same Pfirmann grade and comparison for chondrodystrophic and non-chondrodystrophic dogs, represented by box plots.

Comparison of the MTR values for the same grade between NCD and CD dogs did not reveal any significant differences (Fig 11). For grade I, the mean MTR value was 3047 for NCD dogs, compared with 2873 for CD dogs. For grade II, the averages were 3270 for NCDs and 2807 for CDs. For grade III, the average was 3,014 for NCDs and 3,372 for CDs. Finally, for grade IV, NCD dogs had an average of 3895, compared with 3437 for CD dogs.

**Fig 12:**
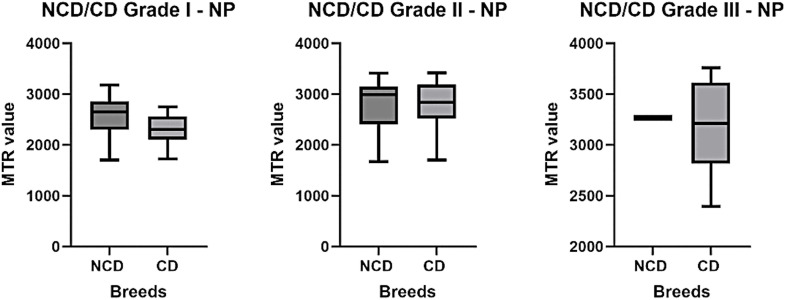
Variation of the MTR for the NP as a function of the same Pfirmann grade and comparison for chondrodystrophic and non-chondrodystrophic dogs, represented by box plots.

Similarly, at the NP level (Fig 12), no significant difference was observed between the two populations for the same grade. For grade I, the mean MTR value was 2577 in NCD dogs, compared with 2309 in CD dogs. For grade II, the average was 2797 for the NCD breeds and 2782 for the CD breeds. However, for grade III, the number of samples was insufficient to allow for a reliable conclusion.

## 4. Discussion

The standard assessment of disc degeneration is based on T2-weighted MRI sequences, using the Pfirrmann classification, which qualitatively grades severity according to morphological criteria: signal intensity, NP/AF distinction, internal structure and disc cranio-caudal length. Although widely used, this method remains subjective and subject to intra- and inter-observer variations. In contrast, quantitative MRI, particularly the measurement of MTR, provides a more objective assessment of disc composition, particularly macromolecules such as collagen. Therefore, this study evaluated the correlations between MTR values and Pfirrmann grade for the whole disc, NP and AF in CD and NCD dogs.

In humans, several studies [10,17] have demonstrated a positive correlation between MTR values and Pfirmann grades, underlining the value of MTR as a quantitative marker of disc degeneration. These observations were extended to the canine model in a study that confirmed its validity in chondrodystrophic (CD) breeds [12]. Consistent with these results, our study confirms a significant correlation between MTR values and Pfirrmann grades, for the intervertebral disc as a whole, but especially for the NP, with particular importance in CD breeds.

Disc degeneration is a frequent and early phenomenon in CD dogs [18]. MRI remains the reference imaging technique for assessing the stage of this degeneration [19] and a study on canine models has shown that MTR enables early detection of traumatic or degenerative changes to discs, these results were confirmed by histological and biochemical analyses [11]. That prior study on CD dogs already demonstrated significant differences between grades I and II; our results extend these observations by highlighting marked differences between grades I and II (***) and II and III (**) in CDs, as well as a more modest difference between grades I and II (*) in NCDs. These results suggest a more rapid and more marked progression of degeneration in CD dogs, in line with their known genetic predisposition [20,21]. This population shows early degeneration [7,22] linked to the premature loss of notochordal cells and the rapid reduction in proteoglycans [5]. The marked transitions between grades I, II and III correspond to major histological changes over a short period of time, which explains the significant variations in MTR, which is sensitive to the density of macromolecules such as collagen. In NCD breeds, degeneration is more progressive, associated with slow remodelling of the extracellular matrix, which explains the small variation in MTR between grades I and II [5,7,22]. However, no significant difference was found in AF for the two types of population. This result is in agreement with the observations of Chung et al (1995), who also reported a stabilisation of the MTR in the AF despite the progression of disc degeneration [23].

Comparison of MTR values for the same grade between populations showed no significant difference, indicating that despite more marked transitions in CD dogs, MTR remains comparable between the two groups at each stage. This result is consistent with several studies that report similar alterations at advanced stages. Degeneration begins earlier in CD dogs, often as early as three months of age, with initial involvement of the NP and then the AF, whereas it occurs later and more progressively in NCD dogs, affecting NP and AF simultaneously [5,6]. Despite these temporal and spatial differences, the histopathological and biochemical changes associated with the same Pfirmann grade converge between populations. Several studies confirm this similarity in tissue characteristics at the same stage, which explains the absence of any significant difference in MTR [5,24–26]. The MTR therefore appears to be a reliable and objective indicator of the degree of degeneration, reflecting comparable changes at the same stage regardless of the population.

Several studies suggest that the MTR sequence is less sensitive than other quantitative MRI techniques for detecting early changes in the intervertebral disc. In particular, T2 and T1 mappings have been shown to correlate closely with histological changes associated with disc degeneration, such as loss of hydration, disorganisation of the extracellular matrix and biochemical changes in the nucleus pulposus. These results were observed in particular in a sheep model [27] as well as in humans at cervicothoracic level, where T2 mapping has been shown to be more sensitive than MTR for diagnosing disc degeneration [17]. Although the MTR is recognised for its ability to reflect changes in collagen, its sensitivity to early stages appears to be more limited according to certain publications [5,17]. However, a recent study carried out on post-mortem canine intervertebral discs highlighted the ability of the MTR to detect early biochemical alterations [12]. In this *ex vivo* context, the absence of movement-related artefacts probably facilitated the detection of these subtle changes, in particular the disorganisation of the collagenous network. These results suggest that MTR performance is highly dependent on the acquisition conditions and the experimental model used. With this in mind, a direct comparison of the performance of the MTR, T1 and T2 mappings, within the same study, and by comparing them with the grades of the Pfirrmann classification, would enable a more precise assessment of their complementarity. Such an assessment, carried out *in vivo*, would be particularly useful for refining the characterisation of the different stages of disc degeneration in the clinical context.

This study presents a pilot methodological investigation assessing the potential of MTR as a quantitative biomarker of intervertebral disc degeneration in chondrodystrophic and non-chondrodystrophic dogs. As a pilot study, the present work was not designed to provide definitive histological validation or exhaustive MRI–histology correlations, but rather to explore feasibility and identify biologically relevant trends to be addressed in future dedicated validation studies. While our results support the feasibility and reproducibility of this approach, several limitations must be acknowledged when interpreting the findings.

A first important limitation concerns the sample size and grade distribution. The study included only nine dogs, with a predominance of discs classified as Pfirrmann grade I and only a single disc classified as grade V. This skewed distribution limits the statistical power and prevents a robust evaluation of MTR performance across the full degenerative spectrum. As a result, the generalizability of these findings is limited, and caution should be exercised when extrapolating them to broader populations or advanced degeneration stages. Future studies with larger and more balanced cohorts will be essential to confirm the trends observed here and to refine potential MTR cut-offs for different degeneration grades.

In addition to sample size limitations, several statistical considerations should be acknowledged. Pfirrmann grades represent ordinal variables, and while correlation analyses allowed the identification of global trends between degeneration severity and MTR values, future studies with larger cohorts may benefit from non-parametric or ordinal statistical approaches. Moreover, multiple intervertebral discs were analyzed per dog, which may introduce non-independence of observations. Although this exploratory pilot study focused on feasibility and overall associations, future validation studies should consider hierarchical or mixed-effects models to account for within-subject variability. In addition, intraclass correlation coefficients could be applied in future studies to more accurately assess inter- and intra-observer reliability. These limitations do not invalidate the present findings but should be considered when interpreting the results.

Another critical methodological aspect relates to the use of frozen cadaveric samples. Freezing and thawing are known to alter tissue water content and macromolecular structure, which may influence MRI-derived metrics such as MTR. Although several previous studies have demonstrated that post-mortem imaging of thawed samples can yield interpretable images [28,29], the potential impact on tissue integrity and quantitative values cannot be ignored. In our study, freezing was applied homogeneously to all the samples, which means that the differences in MTR can be considered interpretable between grades. However we have addressed this limitation by correlating the imaging findings (Pfirrmann grading) with complementary macroscopic and histological assessments, including Thompson grading and staining with Picrosirius red and Alcian blue. Picrosirius red, by binding to collagen fibres, enables the density and organisation of the collagen network to be assessed, particularly in the annulus fibrosus [30,31]. Alcian blue highlights the presence of glycosaminoglycans, major components of the nucleus pulposus, the reduction of which is an early sign of degeneration [18]. This integrative approach provides internal validation of the imaging-based evaluation and increases confidence in the interpretation of the results despite the absence of fresh tissue imaging. Nonetheless, future work should ideally include fresh or minimally preserved tissue samples to better characterize the relationship between MTR and true biological degeneration processes.

Additionally, the absence of advanced biochemical or molecular validation—such as the use of histochemical markers (MMP-3, IL-1β)—represents another limitation. These markers could provide deeper insight into the underlying biological processes associated with MTR changes [32]. Although these analyses were beyond the scope of this pilot study, integrating such molecular endpoints into future protocols will be an important step toward validating MTR as a robust biomarker of intervertebral disc degeneration.

From a practical standpoint, the current MTR acquisition time of 27 minutes poses a challenge for clinical implementation in veterinary settings. While this protocol was optimized for methodological precision, sequence acceleration and optimization strategies—such as parallel imaging, compressed sensing, or the use of reduced field-of-view acquisitions—could significantly reduce acquisition time and improve clinical feasibility [33].

Finally, this work should be positioned clearly as a proof-of-concept study rather than a definitive validation of MTR for clinical use. Our study confirms the validity of the MTR as a quantitative biomarker of disc degeneration in dogs, particularly useful for detecting early changes in chondrodystrophic breeds. Despite certain methodological limitations, the correlations established with Pfirrmann grades support the value of MTR for objectively characterising disc damage. Further work combining quantitative MRI, histology and biochemistry, particularly under *in vivo* conditions, is now required to refine our understanding and monitoring of disc degeneration in dogs.

## Supporting information

S1 File Data dog MTR value. (TXT)

S2 File Data NCD and CD dogs MTR values Grade 2. (TXT)

S3 File Data NCD and CD dogs MTR values Grade 3. (TXT)

S4 File Data NCD and CD dogs MTR values Grade 4. (TXT)
